# Public perception of plant gene technologies worldwide in the light of food security

**DOI:** 10.1080/21645698.2022.2111946

**Published:** 2022-08-22

**Authors:** Woźniak-Gientka Ewa, Tyczewska Agata, Perisic Milica, Beniermann Anna, Eriksson Dennis, Vangheluwe Nick, Gheysen Godelieve, Cetiner Selim, Abiri Naghmeh, Twardowski Tomasz

**Affiliations:** a Bioeconomy and Sustainable Development Team; b Laboratory of Animal Model Organisms, Institute of Biorganic Chemistry, Polish Academy of Sciences, Poznan, Poland; c KWS SAAT SE & Co. KGaA, Einbeck, Germany; d Biology Education, Institute of Biology, Humboldt-Universität zu Berlin, Berlin, Germany; e Department of Plant Breeding, Swedish University of Agricultural Sciences, Alnarp, Sweden; f Department of Biotechnology, INN University, 2318 Hamar, Norway; g Euroseeds, 1000 Brussels, Belgium; h Department of Plant Biotechnology and Bioinformatics, Ghent University, Ghent, Belgium; i VIB Center for Plant Systems Biology, (Technologiepark 71), Ghent, Belgium; j Department of Biotechnology, Ghent University, Gent, Belgium; k The Faculty of Arts and Social Sciences, Sabanci University, Turkey

**Keywords:** Food security, genetic modification, genome editing, plant biotechnology, plant gene technology, public perception

## Abstract

Achieving global food security is becoming increasingly challenging and many stakeholders around the world are searching for new ways to reach this demanding goal. Here we demonstrate examples of genetically modified and genome edited plants introduced to the market in different world regions. Transgenic crops are regulated based on the characteristics of the product in many countries including the United States and Canada, while the European Union, India, China and others regulate process-based i.e. on how the product was made. We also present the public perception of state-of-the-art plant gene technologies in different regions of the world in the past 20 years. The results of literature analysis show that the public in Europe and North America is more familiar with the notion of genome editing and genetically modified organisms than the public in other world regions.

## Introduction

Economic development, a growing world population and changes in dietary habits have resulted in increasing and shifting demands for food. New breeding methods are required to minimize the impact of climate change as the traditional crop breeding methods are time consuming and resource intensive.^1^ According to the State of Food Security and Nutrition in the World 2021 report,^2^ ‘we are not on track to meet our commitments to end world hunger and malnutrition in all its forms by 2030.’^2^ This is caused to a large degree by the frequency and intensity of conflicts, climate variability and extremes, and economic slowdowns and downturns. The State of Food Security and Nutrition in the World 2021 report identified six transformation pathways, singling out the ‘scaling up climate resilience across food systems’ as the one with the particular potential to address it effectively and on a large scale as in the past two decades increased efforts have been made to develop new and improved crop varieties.^2^ However, the legal situation is diverse worldwide and influences the development of plants produced by genome editing (GE).^3^

According to the European Commission Farm to Fork (F2F) strategy, innovations in plant breeding and crop production can contribute to a more sustainable food system.^4^ However, in the case of the EU, the continued uncertainty about the regulatory status of genome edited organisms may be the key obstacle to reaching this goal. On 29 April 2021, in light of the Court of Justice’s judgment in Case C-528/16 on mutagenesis,^5^ the European Commission (EC) submitted a study regarding the status of novel genomic techniques (NGTs) under Union law.^6^ The study addresses multiple aspects related to NGTs. While organisms obtained through NGTs are currently considered subject to the genetically modified organisms (GMO) legislation, it is also acknowledged that there is a lack of key definitions, giving rise to ambiguity and regulatory uncertainty. The EC study regarding the status of NGTs^6^ describes the limitations of the capacity of EU legislation to keep pace with scientific and technological progress, which cause implementation challenges and legal uncertainties. In addition, it may not be justified to apply different levels of regulatory oversight to similar products with similar levels of risk, as is the case for plants conventionally bred and obtained from certain NGTs. Finally, it is highlighted that more effort should be made to inform and engage with the public on NGTs and to assess their views.^6^ Importantly, in the USA, most South American countries, Australia and Japan, certain GE plants are not subjected to GMO regulations.^3,7,8^

The main international and national scientific organizations accept the scientific consensus that food produced from genetically modified (GM) crops is safe.^9,10^ Nevertheless, polarized debates about the use of biotechnology in agriculture, in particular GM food, continue to take place.^11^ GM food is an even more contested topic than evolution,^12^ vaccination or climate change in several parts of the world.^13,14^ Worldwide, consumers are showing limited understanding, misconceptions, and even unfamiliarity with plant gene technologies in agriculture,^15^ including GMO, new breeding techniques (NBTs) and GE. Consumer’s attitudes toward it widely differ, as well as their level of concern or approval of GM food and its safety.^16^ Public perception is one of the critical parameters influencing the development and commercialization of plant gene technologies, which are still controversial for today’s food consumers, especially in the European Union (EU).^17^

Unlike the ‘deficit model’ of early science communication suggests,^18^ it is not primarily lack of education or knowledge that causes controversy or even rejection of science. While the relationship between knowledge and attitudes toward controversial science topics^13^ is assumed to be generally positive,^19,20^ emotions, ideologies, individual norms, and values are described as ‘roots of attitudes’ toward these topics.^21^ Blancke et al. (2015)^22^ described that intuitions and emotions play a major role in the rejection of GMO, with GM foods being more contested than other applications of GMO.^23^ Furthermore, there is evidence that attitudes toward GM food safety are built based on motivated reasoning.^24^

Rose et al. (2020) used a survey with adults from the US Midwestern state to examine how agreement with specific risks and benefits of the technology impacts rejection of GM foods.^25^ The authors noted that GM food rejection is influenced by public perceptions of various salient aspects of the technology, focusing on its potential risks and benefits, as possibly disseminated by the media. According to Frewer et al. (2013), perception of risks and benefits linked with all aspects of GM agri-food application has been increasing over time, independent of whether the target for the use is animal or other GM uses.^26^ Runge et al. (2017) noted the decline in confidence that the federal government can ensure the security of food supplies, although it is unclear whether this is related to increased perceptions of risks related to food or to a wider decline in general confidence in government.^27^

Like the legal situation and state of genome editing that is diverse worldwide, the public perception of plant gene technologies differs across regions of the world. These differences in opinions are not grounded in science, but rather in politics, psychological, social, cultural, personal and economic factors, among others.^28^ This article presents 1) a comprehensive overview of the state of legislation and cultivation of GM and GE crops worldwide and 2) the results of a systematic review of public perception of plant gene technologies in different world regions. Hence, this article provides an extensive descriptive summary of the current situation of plant gene technologies and their public perception over the last 20 years.

## Conceptual Design and Methodology

To investigate the state of the art of public perception of plant gene technologies across the world, a keyword search on the Web of Science was performed. This search was conducted with the keywords from the following groups:

1. acceptance, attitude, opinion, perception;
2. genetically modified organisms/GMO, genome editing (without human), biotechnology, genetic engineering, genetically modified food/GM food;
3. survey.

The condition was that each search included one keyword from each group and all possible combinations of words have been used. Results within the period 2000–2021 have been examined. Only articles in the English language were extracted.

The Web of Science has been chosen as the main source for searching the publications, based on several premises. First, there are high standard research papers, as well as high-influence publications, written in the English language. Second, the Web of Science only includes peer-reviewed journals. Third, this source provides metadata on the document type and the language of the documents. Moreover, there is more control over the search, with advanced search options.

Criteria for searching in the Web of Science are presented in Table 1. Searches included all of the searchable fields using selected queries (see Table 1). Moreover, reports related to the perception of biotechnology, genetic engineering and genome editing in Europe were analyzed [e.g.^29–36^]

**Table 1.** Criteria for searching and their results in the web of science^*.^

Criteria for searching	Results
**GMO**	
Keywords: “perception of genetically modified organisms” and ”perception of GMO” and “survey”	30
Keywords: “opinion of genetically modified organisms” and ”opinion of GMO” and “survey”	18
Keywords: “attitude towards genetically modified organisms” and ”attitude towards GMO” and “survey”	16
Keywords: ”acceptance of genetically modified organisms” and ”acceptance of GMO” and ”survey”	13
**Genome editing**	
Keywords: “perception of genome editing” and “survey” without “human”	5
Keywords: “opinion of genome editing” and “survey” without “human”	6
Keywords: “attitude towards genome editing” and “survey” without “human”	3
Keywords: “acceptance of genome editing” and “survey” without “human”	5
**Biotechnology**	
Keywords: “perception of biotechnology” and “survey”	362
Keywords: “opinion of biotechnology” and “survey”	222
Keywords: “attitude towards biotechnology” and “survey”	133
Keywords: ”acceptance of biotechnology” and ”survey”	200
**Genetic engineering**	
Keywords: “perception of genetic engineering” and “survey”	69
Keywords: “opinion of genetic engineering” and “survey”	41
Keywords: “attitude towards genetic engineering” and “survey”	28
Keywords: ”acceptance of genetic engineering” and ”survey”	35
**Genetically modified food**	
Keywords: “perception of genetically modified food” and “perception of GM food” and “survey”	128
Keywords: “opinion of genetically modified food” and “opinion of GM food” and “survey”	46
Keywords: “attitude towards genetically modified food” and “attitude towards GM food” and “survey”	54
Keywords: ”acceptance of genetically modified food” and “acceptance of GM food” and “survey”	97
**All**	**1511**

*Search steps: www.webofknowledge.com Select a database: Web of Science Core Collections. Basic search. All keywords were searched in the ‘all fields ‘category. Custom year range 2000–2021.

Following the search, all publications were checked in terms of the type of article. Articles from different groups (GMO, genome editing, biotechnology, genetic engineering, GM food) were deleted in cases of duplicates to avoid repetition. Only original research (not review or opinion) has been chosen for further analysis. Articles related to genome editing were analyzed in the field of plant biotechnology (without the topic of human genome editing).

A total of **N = 409** papers were identified from the Web of Science (see Table 2). Results were presented separately for the different continents/regions and based on the subject of interest (GMO, genome editing, biotechnology, genetic engineering, GM food).

**Table 2.** The number of publications by regions.

Region	Number of publications
Europe	127
Asia	100
North America	98
Latin America	19
Africa	20
Australia	20
World	25
ALL	409

To describe the public perception of plant gene technologies in regions, the following criteria have been selected: analysis of the public perception of genome editing, attitude toward biotechnology, GM food, GMO, supporting different applications of biotechnology, risks/concerns toward GMO, GM food, willingness to eat/buy GM food, trust in various stakeholders, labeling of GM products. Each region was analyzed based on those criteria.

Furthermore, a wealth of terminology has appeared along with the development of various biotechnological methods designed to alter the genetic material of different organisms. Some of the terms may relate to scientific jargon and others rather to a legal context. While a generally (and globally) accepted definition has not yet been established for many of them, and while some of the terms are often used interchangeably, certain terms have obtained a legal definition in certain jurisdictions. In this review on public perceptions, the variation in terminology used between the different studies presented a challenge, making comparative analysis difficult. We therefore provided a note on terminology to facilitate and support the discussion about the different survey results (see Glossary).

To describe the current state of legislation and cultivation of GM and GE crops worldwide, authors used materials from the website Global Gene Editing Regulation Tracker.^37^ Additionally, the review of literature from different regions of the world has been conducted to complete the information.

## GM and GE Crops Worldwide and Their Role in Building Global Food Security

To feed the world population from 2050 onwards, world food production needs to increase by 25% to 70%, according to different sources.^38,39^ A potential solution is to increase the acreage of farmable land, however, the vast majority of it is already used for various types of agriculture.^40^ According to World Bank data, around 36% of the world’s total land area (about 12.9 billion ha) in 2018 was considered agricultural (4.7 billion ha), while 10.8% of land area was considered arable^a^^a^Agricultural land refers to the share of land area that is arable, under permanent crops, and under permanent pastures. See also: https://ec.europa.eu/eurostat/statistics-explained/index.php?title=Glossary:Agricultural_area_(AA)https://ec.europa.eu/eurostat/statistics-explained/index.php?title=Glossary:Arable_land.
^41^ The role of GE and GM crops for food security is the subject of public controversy.^42–44^ As noted by Ricroch (2019), the new applications of GE technology in plants in agriculture will change our everyday lives due to many benefits of GE technology, such as reducing food waste.^45^ The development of GE crops could contribute to food production increases and thus higher availability of food, as well as its increased nutritional value. Other benefits cover the reduction in pesticide poisoning, lower cancer incidences, decrease in the number of farmer suicide occurrences and increased farmer mental health benefits.^40,46–77^ Moreover, the economic benefits (of US$167.8 billion) reached by 18 million farmers worldwide between 1996 and 2015 resulted from the increase in yield, production gains, and cost savings of GM crop cultivation.^48^

The key goal of GE and GM in agriculture is to develop crops that are resistant to abiotic stresses, emerging pathogens, that can help reduce pesticide use, and have increased nutritional values.^40,49–58^ According to ISAAA (International Service for the Acquisition of Agri-biotech Applications), to date a total of 44 countries granted regulatory approvals to 436 GM events (either stacked or singular traits), covering 33 plant species and 44 GM commercial traits for use in food, feed and/or for cultivation.^59^ The most widely targeted traits are herbicide tolerance (359) and insect resistance (307), followed by modified product quality (99), pollination control system (31), disease resistance (29), abiotic stress tolerance (12) and altered growth/yield (4). The most widely modified plants (by events) are maize (*Zea mays*) – 152, cotton (*Gossypium hirstum* L.) – 66, potato (*Solanum tuberosum* L.) – 50, soybean (Glycine max L.) – 40 and Argentine canola – (*Brassica napus*) – 39.^59^

**Figure 1.**
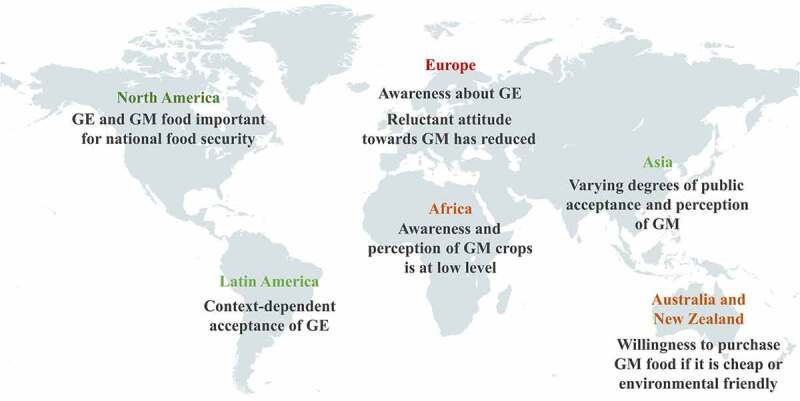
Highlights of GM crop cultivation worldwide in 2019.

In total, in 2019, 190.4 million hectares of biotech crops were grown in 29 countries. The most adopted biotech crops by the 29 countries were soybeans, maize, cotton, and canola^60^ (see Fig. 1). The top countries that cultivated GM plants are presented in Fig. 1. Forty seven countries in Africa currently cultivate GM crops, with South Africa being the largest GM crop producer in Africa.^60^ The examples of recently developed GE and GM products from different parts of the world are presented in this section.

### North America

The US has a dynamic history of development, commercialization and consumption (use) of GM plants (and recently, animals). The first GM plant, tomato, was developed in 1982 and by 1996, FlavrSavr Tomato was the first GM plant variety available for commercial use in the US^61^⁠. The US and Canada were among the first countries to take concrete regulatory decisions upon the regulatory status of several new plant breeding innovations.^62^ They have a strong incentive for it: the US leads the biotech crop planting at 71.5 million hectares and more than 90% of US corn, cotton, and soybeans are produced using GE varieties.^60^ The slope of its progression and general public acceptance is in large part due to the established transparent and effective system for regulating, directing and monitoring the safety of the implementation of genetically engineered plants.

Notably, in the US new GM varieties are constantly being approved for consumption and production and US farmers are growing the first GE plants; some of the examples are given below. In 2015 RNA interference was used to silence polyphenol oxidase production in non-browning potatoes, which were approved by the United States Department of Agriculture (USDA).^63^ Since November 2017, GM non-browning apples^64^ have been sold in the US supermarkets. The ODM (oligonucleotide‐directed mutagenesis) based GE canola cultivar (Cibus) is marketed in the US without any formal approval.^65^ CRISPR–edited *Camelina sativa* (false flax) containing an increased content of omega-3 fatty acids in oil was permitted to be cultivated and sold free from regulation, as the USDA has given it a free pass.^66^ In fall 2018, a soybean with modified oil composition was harvested at a small scale as the first TALEN-based GE crop (Calyxt). Later it was commercialized as High Oleic Soybean Oil – a high-quality food ingredient.^67^ Another company (Yield10 Bioscience Inc.) is planning to conduct field trials with the first CRISPR/Cas edited canola.^68^

The government department Health Canada has been assessing GM foods for more than 20 years. The commercial sale of food derived from the GM rice line known as golden rice was approved in Canada in March 2018^40^ and non-browning potato was approved for sale in Canada in 2016^69^ (full list of approved GM events in Canada are given in the ISAAA database.^70^ As of 2019, over 140 GM foods have been allowed for sale in Canada.^71^ There is no mandatory labeling for GM foods, although voluntary labeling is permitted as long as claims are not misleading.^72^ Canada took a specific stance on GE by regulating any products that contain novel traits, including GE crops, regardless of the process used to develop the product.^73^

### Latin America

Latin American countries cultivated 44% of the global GM crop area. In 2019, both Brazil and Argentina ranked in the top five GM cultivating countries.^60^ Interestingly, eight countries in Latin America (Brazil, Chile, Colombia, Ecuador, Guatemala, Honduras, Paraguay, and Argentina) have already established criteria to determine the regulatory standing of NBTs.^62^ Several Latin American countries also conduct research to develop their own GE food and crops. In Brazil, an antioxidant-rich tomato with high levels of lycopene was developed.^74^ Researchers from the University of Costa Rica, in collaboration with scientists from other countries, are conducting gene editing in rice, especially for drought resistance, as a way to mitigate climate change effects and contribute to food security.^75^ Researchers from Argentina used GE techniques to develop potatoes that do not turn brown and to create hypoallergenic milk.^76^

On the other hand, Ecuador, Venezuela, and Peru do not permit commercial cultivation of GM crops in their territories.^77^ In Ecuador, the import of GM food is permitted as long as it is labeled. Despite the Ecuador GM-free declaration,^78^ in 2019 a Decree 752 was implemented, establishing a product-based regulatory framework for “genetically improved organisms” and deciding that organisms without foreign genes in the final product are exempt from risk assessment.^77,79^ In December 2020, the Mexican President issued a decree banning all imports and approvals of GMO corn. Before this decree, Mexico was one of the world’s largest importers of GMO corn and soybean.^80^ The country wants to withdraw GM corn from human consumption by 2024.^81^

### Europe

In Europe, only one biotech crop – GM maize – was grown on just 102 367 hectares in Spain and Portugal in 2020.^82^ In 2021, EC has authorized the import of seven GM crops (3 maize, 2 soybeans, 1 oilseed rape, and 1 cotton) and renewed the authorization of two maize and one oilseed rape for food and animal feed use.^83^ Since 1996, 109 GM events have gained approval in the EU (ISAAA GM approval database), hence it does benefit from the import of GM crops (mainly soybean, canola, maize, and cotton), however, GM cultivation is still prohibited in the majority of the EU countries.^60,65,77,84^ Moreover, all products containing more than 0.9% of an approved GMO require a label. Outside the EU countries, so far, the Food Safety Authority in Norway has not approved any genetically engineered crops for food or feed. A proposal from 2018 from the Biotechnology Advisory Board in Norway suggested that GE crops without foreign genes do not meet the definition of transgenic GMOs and should be regulated as conventional crops, however, no unique regulations have been proposed so far.^85^ On the other hand, after Brexit, the government of the UK (Department for Environment, Food and Rural Affairs – Defra) proposed removing the current legal barriers to GE crops by the end of 2021, thus enabling GE crops and animals to be imported and cultivated. In a first step, legislative change was adopted for researchers wishing to conduct field trials in the UK with certain GE plants (subject to the category of “higher qualified plants”) are no longer required to submit risk assessments.^86^ However, they will still need to register their field study through a notification procedure.^87^ The next step will be to review the regulatory definitions of GMOs, to exclude organisms produced by GE and other genetic technologies if they could have been developed by conventional breeding for cultivation and market release.^88^ Nevertheless, the regulations have attracted a lot of criticism and concerns from the Lords Secondary Legislation Scrutiny Committee. One of the concerns was that there exists no published guidance on the “scientific and regulatory criteria that will be used to determine whether a genetic change could have occurred naturally or through traditional breeding methods.”^89^ Also, the Committee had concerns about cooperation between researchers in different parts of the UK.^89^

In April 2022, in the UK a field trial of GM and GE barley with boosted expression levels of the *nsp2* gene responsible for interaction with mycorrhizal fungi began.^90^ The aim is to verify whether enhancing the natural capacity of crops to interact with common soil fungi can contribute to more sustainable, equitable food production, to reduce dependency on synthetic fertilizers and promote soil health. Further, it can help farmers in developing countries to reduce production costs and increase their income.^91^

### Africa

Africa currently remains the region with the greatest potential to adopt GM crops. South Africa cultivated 2.7 million ha of maize, soybean, and cotton, while cotton was also grown in Sudan (236.200 ha), Malawi (6.000 ha), Nigeria (700 ha), Eswatini (401 ha), and Ethiopia (311 ha), for a total of 2.9 million ha.^60^ Nigeria has emerged as a leader in the adoption of GM crops. The government has allowed the cultivation of Bt-cowpea in 2019 and has now approved the release of drought tolerant and insect resistant maize (TELA).^92^ In December 2021, Nigeria’s National Biotechnology Development Agency (NABDA) and the USDA implemented a nutrition outreach program aimed at increasing the consumption and demand of GM Bt-cowpea in the country. The program seeks to demonstrate the food safety and nutrition effects of Bt-cowpea, Nigeria’s first biotech food crop.^93^ After nearly 10 years of moratorium, Kenya has recently started National Performance Trials (NPTs) for the TELA maize in preparation of cultivation and approved NPTs for virus-resistant cassava. This reveals a changing attitude toward GMOs in the Kenyan government. Furthermore, the National Biosafety Authorities of both Nigeria and Kenya have recently published guidelines for GE, in which they conclude that if no foreign DNA is present in the final plants these GE plants are not GM.^94,95^

Kenya banned all GMO imports and discontinued all processes toward the cultivation of GM crops after the publication of Seralini *et al*. (2012),^96^ despite that the research institutes in the country had developed multiple useful GM crops. Also in Uganda, a diversity of promising GM crops that have been field-tested,^97^ cannot be transferred to the farmers due to political hesitation.

### Asia

The first GM crop in Asia – a virus-resistant GM tobacco – was commercialized in 1992.^98^ Among Asian countries, India (11.9 million ha), China (3.2 million ha) and Pakistan (2.5 million ha) made the most significant contribution to the cultivation of GM crops in 2019.^60^ Since 2002, China has been a significant importer of GM products with over fifty varieties currently approved.^99^ In recent years, Japan and Australia have issued and refined their implementing regulations and made the first decisions regarding the status of several products. In September 2021, Japan accepted a gene-edited red sea bream named “madai,” which has 20% more meat through applying CRISPR technology. Contrary to GM food – GE products are not subjected to safety screening in Japan.^100^ Recently, researchers from Tsukuba University used CRISPR to develop a tomato with higher content of the neurotransmitter GABA that might help lower blood pressure.^101^ Scientists from the University of Tokyo used a technique called “mitoTALENs” to develop high-yield strains of rice and canola.^102^ In July 2021, the Philippines approved Bt-eggplant (GM variety resistant to eggplant fruit and shoot borer, the most destructive pest of eggplant) for cultivation for direct use as food, feed or for processing. According to a recent study, the commercialization of Bt-eggplant will increase marketable yield by 192% and reduce pesticide application per hectare by 48%.^59,103^ Moreover, the Philippines approved the nutrient-enriched Golden Rice, with additional content of beta-carotene, for planting. This new variety has already received food safety approvals from regulators in Australia, New Zealand, Canada, and the US, but the Philippines were the first to approve its commercial cultivation. Golden Rice is also now undergoing the final regulatory review in Bangladesh.^104^

Political leaders are instrumental in the implementation of new technologies such as GM food crops. A clear-cut example is Bangladesh where prime minister Sheikh Hasina and the agricultural minister Begum Matia Chowdhury embraced GM crops as an important element for economic and food security in the country. Bt-eggplant cultivation in Bangladesh started with 20 farmers in 2014 and has since steadily been increasing, with already 65,000 farmers recorded in 2020–2021, resulting in a significant reduction of pesticide costs, higher yield and thus increased profit for the farmers.^105,106^ The government of the neighboring country – India, where Bt-eggplant was originally developed and initially approved, completely stopped the route to cultivation mainly due to pressure from environmental lobby groups such as Greenpeace.^96^ The publication of Seralini *et al*. (2012) that claimed that GMOs cause cancer strengthened the psychology of fear. The retraction of the paper on the basis of inconclusive data could not reverse the damage done. As a result, Bt-eggplant is still today under moratorium in India.^96^

### Australia and New Zealand

In Australia and New Zealand 142 and 113 GM crop events, respectively, have been approved to date.^107^ In Australia, GM cotton, canola and safflower were cultivated under 0.6 million ha.^62^ Moreover, Australian GMO crop field trials cover 14 sites and an area of nearly 7 ha, including 12 sites (chickpea, wheat, sorghum, cotton) on post-harvest monitoring stage, and two currently ongoing banana trials.^108^ In Australia, if the product does not contain foreign DNA, then it is not regulated as a GMO.^109^ To date no GE crops have been approved in Australia, except for one type of GE crop obtained using SDN-1 type genome-editing technology that has been deregulated as a conventional variety.^62,109,110^ Interestingly, researchers from Murdoch University recently developed a low gluten index potato using CRISPR.^111^

Contrary, in New Zealand all types of GE crops are regulated as GMOs.^112^ According to a report from October 2021 in New Zealand no organization has submitted an application for a conditional or full-scale release of a GE plant, however this country permits the import of GE food products, based on 84 GE events that have been approved by Food Standards Australia New Zealand (FSANZ). These food products may be for either direct human consumption or for animal feed.^113^ Examples of species relevant to New Zealand’s plant-based primary industries that have been modified using GE technologies including woody species like apples, kiwifruit, grape, sweet orange or poplar, forage crops such as Alfalfa and vegetable crops such as tomatoes, potatoes, cucumber and lettuce have been described in.^114^

## Public Perception of GM and GE Products Worldwide

Since the release of GMOs on the market, many surveys on the public perception of genetic modification and similar technologies have been conducted worldwide. Scientific breakthroughs in GE have boosted its popularity and revitalized the public’s awareness of biotechnology. International experts agreed on the potential benefits of GE crops in terms of agronomic performance (disease resistance, drought tolerance, etc.), final product quality (nutrition, shelf life, etc.), climate change resilience, and global food security.^115^ Surveyed experts believed that health and safety regulations, followed by export trade rules, consumer acceptance, and engagement of the media, all play major roles in determining where and how NGTs are developed and used in agriculture.^115^ International experts highlighted that if GE crops are regulated as GM crops, the cost and time for approval will significantly increase.^116^

**Figure 2.**
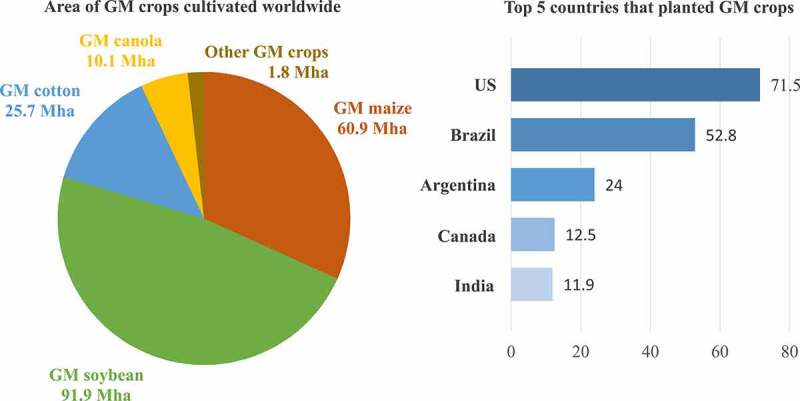
Public perception of plant gene technologies across different regions in the world.

Consumer behavior is influenced by psychological, social, cultural, personal and economic factors, including scientific knowledge, lifestyle, personal welfare, income, religion and beliefs, and consumer perception.^117^ Their attitudes about GM food are complex, vary worldwide and are often influenced by affective factors like intuitions, emotions and values (e.g., purity;.^22,118^) In the last two decades, the products of plant gene technologies have been adopted at different paces across different regions of the world (see Fig. 2). In this study, the authors determined the current status of the public perception of plant gene technologies in the world through a systematic literature analysis of published surveys and discussed the trends in different regions of the world over the last 20 years. Additional information and references are presented in the Supplementary Table 1.

### North America

Genetically altered products in the form of crops and food ingredients have been on the US market and widely consumed for more than two decades, which is in itself a sharp distinction to the situation in most parts of the world and reflects a wider public consensus regarding the stated technologies. Approximately 69% of the population agreed, and only 22% disagreed about whether the application of biotechnology in crops should be encouraged. With regards to its application in food, 58% of US respondents strongly encouraged this application, while 33% disagreed.^119^

For US consumers, GM crops meet many of their needs. National food security is higher rated than consumer-related (nutritional characteristics) or producer-related benefits (production efficiency). Profitability is a high priority for both Canadians and Americans, who believe that NBTs make food more affordable.^120–122^ US citizens reported 5% higher concern about the issue of GE in food relative to the 17 other issues in biotechnology in the series of 47 monthly surveys conducted in the US (N > 1000).^123^ US farmers, the primary consumers of GM seeds, felt confident that GM crops had undergone thorough scientific risk assessment by the authorities and did not perceive human and animal health to be an issue.^124^ They were aware of the environmental risks and complied with the government guidelines with regard to optimal weed control (87% of farmers) and insect refuge (82% of farmers) in Bt corn.^124^

One of the major concerns in US society is the perception of the fairness of the distribution of benefits. US and Canadian consumers believe that most of the benefits of NBTs go to the private sector. Chemical suppliers were perceived to receive the largest benefits (18%), governments, food processors and farmers shared approximately equal benefits (15%, 15% and 14%, respectively), and consumers (11%) and universities were perceived the least (9%).^120,123,125,126^

Americans perceive GM crops as overall beneficial^120^ in terms of food availability: they were first developed for their agronomic benefits, which translated to the economic benefits, and marketed as such. The belief that modern plant breeding makes food more affordable is well founded, and it is obvious that agricultural innovations have had a large impact on food availability in the US: in 1930, American households spent 21% of disposable income on food, while this figure fell to 5.7% in 2012.^127^ One of contributing factors has been the introduction of genetically engineered crops: comparable figures for countries that have rejected genetically engineered crops, such as Germany and France are 10% and 13%, respectively^120^⁠.

The US citizens trusted most in academic scientists and farmers and least in retailers and food manufacturers. In the US, government agencies and environmental organizations had an average degree of trust in this hierarchy^128^ because citizens perceive government agencies as having dual roles in supporting the agricultural community and ensuring food safety for American consumers. The two positions are, at times, in conflict.

Under the 2016 law, the US requires labeling GMO and genetically engineered food as “bioengineered food” (BE) for its disclosure, with a mandatory implementation date of January 1st, 2022.^129^ However, it is still unclear who will bear the costs of the segregation measures and the potential identity preservation and traceability of the produce in connection with exports and/or domestic consumption.^124,125,127^ The primary concern of producers was that mandatory labels might signal that genetically engineered food is unsafe for health or harmful to the environment.^125^ The US consumers have been unequivocal about their wish to be able to distinguish between BE and non-BE food^28,125^ despite their general approval for the technology^119^ and willingness to buy and consume GM products.^130^ Recent studies revealed that BE disclosure will not have adverse effects on the acceptance of genetically engineered food, and they might in fact do the opposite: in Vermont, the opposition toward genetically engineered food decreased relative to the other states in the US when mandatory labels were implemented.^125,131^

US consumers appear to welcome products of GM and NBTs⁠^115^ and are willing to pay 19–26% premiums^125^ if transparent information is shared^131^ and if specific needs, such as improved nutrition^132^ are satisfied. More than 56% of respondents among US consumers were willing to buy and consume both GM and GE food.^130^ Canadian consumers were unlikely to buy a GM product at a higher price compared to a non-GM one, even if it had a better nutritional profile.^133^ They (39%; N = 506) were quite price-sensitive as they indicated that they would likely and very likely buy a nutritionally enhanced GM product if the price were the same as a non GMO product.^133^ This is an interesting result, because it would suggest that what really drives consumer’s purchasing decisions is not the production method but the price of the product.

### Latin America

Studies on public perceptions of GMO or GM and GE food have been performed in only a few countries of Central and South America. The majority of Jamaicans (92%; N = 128) perceived genetic engineering as unsafe.^134^ In Costa Rica, the general public expressed acceptance (> 80%) of GE for purposes such as nature conservation, curing diseases or crop improvement.^135^ Studies in Latin America imply a context-dependency of acceptance: Brazilian high school students showed higher acceptance of GM for producing medicines and vaccines (87%) than for crop (81%) or food (66%) applications.^136^ In Jamaica, 58% of respondents were in favor of genetic engineering to enhance crop plants.^134^

Consumers from Costa Rica perceived potential benefits of GE: an increase in national crop production (66%), improvements in the national economy (64%) and benefits to the environment (57%) and families (61%).^135^ Approximately half of Costa Ricans perceived low or no risk of GE food for health and the environment.^135,137^ In addition, 21% of Costa Ricans expressed fear that GM food poses health risks.^137^ Different crucial stakeholders (N = 52) in Mexico did not perceive high risks of GM food consumption but were concerned about the potential impacts on the country’s biological diversity.^138^ More than 60% of Mexican teachers (N = 362) believed that GM foods can help prevent world hunger, 39% considered it to be risky for future generations, 35% thought that GM food can be harmful and 46% expressed concerns that it threatens the natural order of things.^139^ About 68% respondents (N = 1207) from Paraguay reported that they had heard or knew about genetically engineered crops, however, over half of them believed that they are dangerous.^140^

Consumer perceptions concerning consumption of products of genetic engineering were the most surveyed topic in Latin America. Seventy-one percent of Costa Ricans (N = 1018) were in favor of consuming food developed by CRISPR/Cas9 if the nutritional quality was better and 61% if it was cheaper than the conventional product.^135^ Approximately half of Costa Ricans^135,138^ and Jamaicans^134^ would consume food obtained from GE/GM plants if the price was the same as for conventional products.

According to Chilean supermarket customers, the presence or absence of GM in the food and the introduced trait were more important than the price or brand of the product in the decision to purchase.^141–143^ The absence of GM products in oils (87%), as well as milk and tomato sauce (74%), was desirable for the majority of Chilean consumers.^141,142^ However, a high proportion (73%) would accept oils from GM products that reduce the use of pesticides. Approximately 27% rejected all types of genetic modification.^142^

Among Brazilian society (N = 550), approximately 63% qualified the governmental food surveillance activities as weak, and only 3% trusted the current control to be efficient.^144^ Generally, in all studies that investigated the preference for food labeling in Latin America, food labeling was highly favored.^134,136^

### Europe

Based on public opinion analysis, in the last 20 years in the EU, there was a small but significant shift in attitudes toward biotechnology. As noted by Woźniak *et al*. (2021), European society has noticed the benefits of using biotechnology in medicine to prevent or cure diseases and prevent disabilities; however, GM products (especially GM plants) have been accepted to a lesser degree.^17^ Other surveys demonstrated a decrease in reluctant attitude toward GM food by Europeans from as high as 86% in 1999 and 66% in 2010 to 60% in 2019.^145^

The analysis of GM attitudes in three post-soviet countries, the Czech Republic, Russian Federation and Ukraine (N = 382), showed that young adults were more positive in that respect than their parents. The most negative attitude with regard to GM crops was reported in the Russian Federation.^146^ Based on the literature analysis, it is conspicuous that Nordic countries (such as Finland, Sweden, Denmark and Norway) stand out in terms of attitudes and perception of GM food, GMO, and GE. This may result from the early adoption of plant gene technologies in this region.^147^

In Norway, most consumers (N = 2016) were positive about using GE in Norwegian agriculture and aquaculture for purposes that are perceived to promote societal benefit and sustainability, such as reducing pesticide use and preventing crop losses (68% of respondents), supporting climate adaptation of crops (65%), and improving animal and fish health (58–60%).^35^ In Sweden, almost 50% of respondents (N = 992) supported improvement of plant production.^34^ A recent survey revealed that 69% and 54% of Swedish men and women, respectively, had positive or neutral attitudes toward GMOs. This study speculated that the generally high trust in the food system may play a role here.^148^ In Poland, in surveys conducted between 2014 and 2018, higher yields of crops, resistance to pathogens, and increased resistance to drought received the highest scores, while reducing hunger in the world and the use of GM technologies to produce medicines were assessed on average as the least likely advances.^16^ Moreover, in the most recent survey (2019) conducted in Poland every third respondent believed that the use of modern biotechnology in food production, e.g. to increase the protein content, extend the shelf life, or change the taste, is useful (33%), should be supported (31%) and can be accepted (32%). Moreover, 61% of respondents said that using new methods of biotechnology and genetic engineering in the production and processing of food may involve a risk to human health or the environment. On the other hand 69% of respondents said that using microorganisms to treat sewage and other wastes should be carried out and supported.^149^

In the UK, approximately 45% of respondents agreed with the statement that GE opens up new opportunities to tackle global challenges.^36^ However, a large proportion of respondents (46%) said that GE carries too many risks. Although scientific research has not demonstrated that the production and consumption of GM foods is dangerous, consumers are still uncertain about their safety.^36,150^ In Italy in 2019, over half of the general public (N = 1006) (54%) and the majority of scientists (81%) believed it is safe to eat GM foods. Moreover, 64% of scientists (compared to 58% of the general public) believed that GM crops are able to increase food supply due to the higher yield.^150^

Approximately 40% of European respondents were likely to try NBT products.^28^ As reported by,^151^ on average, 36% of all the participants from Belgium, France, the Netherlands, Spain and the United Kingdom (N = 3002) were willing to consume a GM food product, with values ranging from 23% in France to 47% in the UK. Other studies showed that 49% of French consumers (N = 1109) were willing to purchase biotechnology-produced fresh fruit. Factors like environmental awareness, self-reported healthiness, and habits of eating away from home, have been found to enhance the willingness to purchase biotechnology produced fruit.^152^ In an Italian study consumers did not support fungus-resistant grapes (FRG) wines generated from GE hybrids. The responders expressed a premium price for horticultural FRG wines (+9.14%), while the price discount for GE hybrids was – 21.13%.^153^ However, their willingness to buy wine from GE grapes increased after receiving information on the reduction of pesticides used for GE hybrid grapes cultivation compared to non-GE.^153^

More than three-quarters of respondents from Sweden, Denmark and Finland trusted national authorities for information on food risks.^33^ In contrast, less than half of respondents trusted the national authorities in Croatia, Poland and Bulgaria and France.^33^ Most Norwegians (70%) trusted that GE products developed and approved in Norway are safe and beneficial to society.^35^ Interestingly, in Bosnia and Herzegovina, 64% of citizens said that the government should allow GM food.^154^ In contrast, nearly half of respondents in Serbia had no trust in the government authorities. They did not believe that the authorities would take into account their interests in future GM food legislative decisions.^155^

Approximately 92% of Poles (N = 1021) demanded that GM food products be labeled.^156^ In another study conducted in the UK and Poland (N = 976), over two-thirds of the people surveyed supported obligatory labeling of GM food.^157^ For 76% of Norwegians and 87% of Spanish respondents, labeling was important.^35,158^

### Africa

In African countries, no perception studies on GE have been performed, but a fair number of articles on GMO perception are available, all focusing on GM crops and food. A survey with South African respondents (N = 3500) revealed that 41% agreed that GM food is not compatible with religious beliefs and 30% thought the genetic modification of food was wrong.^159^ Overall, a more positive attitude toward GM food was associated with the perception of benefits being higher than of risks. In Uganda, 86% of farmers would grow GM maize (drought tolerant, insect resistant or both) because they perceived it as an opportunity for lower yield loss.^160^ When confronted with specific examples, such as GM maize or banana, that are either healthier or cheaper or require fewer pesticides, willingness to buy was high (68–92%) for consumers in Kenya,^161^ South Africa^159^ and Uganda^162^ but not in Tanzania (<40%).^163^ Most Ghanaian farmers would choose non-GM seeds if given the choice between non-GM or GM seeds. They were more concerned about public acceptance than about the possible risks of GM crops.^164^

The trust of Ugandan consumers in controlling GM crop release was the highest among local leaders (78%) and some ministries (NEMA 89%), followed by scientists (NARO 73%, university 66%).^162^ Trust in NGOs and food processors was lower (62% and 41%, respectively). Cluster analysis showed that trust in the government correlated with a more positive attitude toward GM crops.

The 2016 survey in South Africa revealed that 75% of respondents agreed with the statement that food products containing GM should be labeled.^159^

### Asia

Asian countries, with 59.76% of the world population, show varying degrees of public acceptance and perception about plant gene technologies.^165^ In China^166^ and South Korea,^167^ well-educated people (college degree or higher) were more likely to engage in deliberate reasoning when shaping their support and were more skeptical toward GM foods. In Japan, experts in molecular biology showed the highest benefit and the lowest risk perceptions compared to experts in other fields and laypeople. It was also found that laypeople’s attitudes revealed the influence of scientific literacy on attitudinal change toward crops developed with new breeding technologies for benefit perceptions but not risk or value perceptions.^168^

In China, about 40% of respondents (N = 596) perceived GM foods as safe, 26% perceived them as unsafe, and 35% did not know whether GM foods are safe. About 73% of consumers believed they have eaten GM foods without being aware of it. Interestingly, 79% of consumers indicated they intend to purchase GM food.^169^ The mandatory labeling “contains GMO” and voluntary labeling “non-GMO” exist concurrently on the Chinese market. Consumers considered both the “contains GMO” and “non-GMO” labels important (89% and 83%, respectively).^169^

South Korean residents (N = 450) who had more information about GM foods, regardless of their income, preferred traditional food and were more likely to pay higher prices for GMO labeling policies.^170^ Other studies showed that 36% of South Koreans (N = 1003) were willing to purchase biotechnology-produced fresh fruit (they had the highest rate among studied countries).^152^ In Turkey, 80% of students surveyed (N = 670) showed a strong desire to label GMOs, and 65% believed the information on the food package was not convincing.^171^ In Singapore^172^ and India^173^ attention to food safety and novel food news was associated with public support for labeling, and consumers’ benefit perception was significantly associated only with public support for banning novel foods. In Pakistan, 70% of students (N = 400) said that the food should be accurately labeled and the decision should be made at the consumer’s end.^174^ The findings of a study conducted in China^175^ showed that 57% of the respondents expressed the need for traceability, 66% perceived the nutrition benefit, and 63% perceived the health risk of GM soybean oil. Moreover, most consumers (72%) trusted in the agency overseeing GMO safety.

In Turkey,^176^ China,^177^ Japan,^178^ and Malaysia,^179^ the minority of consumers approved of the promotion of genomic studies and biotechnology related to GM food/crops. In Asia, farmers were more receptive to GM crops as they are the direct beneficiaries of this technology. Insect-resistant GM (Bt) cotton farmers in China showed a very positive attitude, as Bt-cotton provided them with significant economic benefits.^180^ A survey among the Bt-brinjal (eggplant) farmers in Bangladesh showed that 97% of farmers believed that Bt-brinjal cultivation reduced pesticides, 96% believed it reduced the concern of insecticide use, and thus, 96% considered Bt-brinjal safer for human health.^181^ According to Shelton *et al*. (2020), about 80% of Bt-eggplant farmers were satisfied with the yields and the quality of fruit. Three-quarters of Bt-eggplant farmers were willing to plant Bt-eggplant next season because of the achieved benefits of higher yields, revenue and fruit quality. Many farmers highlighted the benefits of reduced insecticides usage.^105^

Studies conducted in Malaysia pointed to the significant effect of religion, as one of the important background variables, on the ethical perception of modern biotechnology.^182,183^ Malaysians (N = 434), the majority of whom were Muslim, did not consider modern biotechnology very threatening to the natural order of things (54%). They acknowledged the high benefits that modern biotechnology could bring to society (75%).^182^ A survey in Iran (N = 210) emphasized that the level of religiosity and moral and ethical beliefs were the most powerful predictors of social risk perception.^184^

### Australia and New Zealand

In New Zealand, consumers were not willing to buy GM food (90%)^185^ unless there were clear environmental benefits (63%).^186^ In Australia, more research has been performed recently, with even a survey on the perception of GE.^130^ In this survey, the attitudes toward herbicide-resistant rice were very similar regardless of whether the rice was GE or GM.

Australian high school students were highly supportive of biotechnology for engineering microorganisms and humans.^187^ More than half of students (N = 465) also supported GM plants. In another Australian study,^188^ high school students rated biotechnology for medical applications higher than other domains, such as the use of GM plants. In the Australian surveys, a more negative attitude toward GMOs was commonly associated with a higher perception of risks than benefits.^189^ Nevertheless, the risk of GMOs was perceived to be lower than that of additives or pesticides.^190,191^

Australian farmers were aware of GMOs and were interested in cultivating GM pulses (grain legume), especially at higher yields. They were also willing to consume GM wheat.^192^ Australian consumers were only willing to purchase GM food if it was cheap.^191^ However, approximately three-quarters of young people (high school students) believed that GM crops have benefits and therefore supported them.^187,188^ Recent surveys on GM rice revealed that 69% of surveyed persons were willing to eat insect-resistant GM rice,^193^ while this percentage was approximately 60% for herbicide-resistant rice.^130^

Less than 20% of high school students trusted the press and the internet, despite the latter being their most important source of information.^188^ On the other hand, researchers received trust from more than 80% of students, while government, farmers and environmental organizations scored low (25–30%).

## Concluding Remarks and Future Perspectives

The results of the public perception of plant gene technologies showed that among all regions of the world the European public seems to hold the strongest negative attitudes toward GM foods (though with significant regional differences) and very rigorous regulations concerning GMO and GM food/feed.

In the EU, where the most strict GMO/GE regulations are in force, the acceptance of this technology in agriculture and food production is among the lowest. The societies in North and South America, global leaders in the development and commercialization of GM crops, support GM and GE to a higher degree. Global GMO policy shows divergence, not convergence. Two extremely contrasting approaches to the GMO legislation matter are represented by the US as strong advocates for GMOs through approval and production, and the EU where the precautionary principle to GMOs is being applied. Other countries are mostly somewhere in between the liberality of the US and the rigorousness of the EU, with noticeable differences among countries’ policies. By its nature, law consists of a number of norms which constitute obligatory rules of behavior for the members of the society. These legal norms are closely related to various social values (the core beliefs about what is moral and immoral, good and bad, acceptable and unacceptable), norms (the “action aspect” of values), folkways (customs that guide daily interactions and behaviors) or mores (deeply and intensely norms on what is right and wrong), that serve in a direct or indirect manner.^77,194,195^ Importantly, in the US, and most South American countries, such as Argentina, Brazil, Chile, and in Australia, and Japan, certain GE plants are under no GMO regulation.^3,7^ The European Commission is taking action to rebuild the current EU GMO legislation for excluding NGTs from the current detection, labeling, and approval requirements.^196^ It is quite important to inform the public about the benefits of NGTs and to break the resistance to GM crops. In Germany, in the position paper from 2019, overcoming public resistance against genetic engineering in food production was prioritized.^197^

According to Lougheed (2009) nowadays scientific expertise and analyses play a more significant role in defining societal values than ever before and thus exert changes to the law. Undoubtedly, shaping the law on scientific technologies like GM and GE crops relies also on other factors including bureaucratic, political and societal interests.^198^ The evolution of the law on GE crops demonstrates that it is not constant and must accustom to the mores of society, and experiences of 25 years of cultivation and regulation of GM crops.

The results of the study showed that the level of education has a great impact on public perception of plant gene technologies. As seen in Japanese and Spanish studies, people with a background in molecular biology or biology have a much more positive perception of GM and biotechnology than laypeople. Moreover, the experts’ opinions about GM food/crops, GE or biotechnology were more positive than those of laypeople. We also found out that the public in all regions of the world agreed that labeling GM products is important and necessary since consumers want information about the kind of genetic technology applied to produce food.

This study’s findings must be contextualized by the limitations of the underlying studies. Several aspects made the analysis of the literature data on public perception of plant gene technologies difficult to conduct: analyzed surveys varied in terms of the respondents (e.g., experts, students, consumers, general population, farmers), the GM/GE context (e.g., food consumption, medical supplies) and survey methods (e.g., internet, phone, face-to-face interview). Also, how survey questions were formulated had a great impact on the results. In many cases, surveys were conducted using different methods. The lack of standardized survey methods limits direct comparability of results, as it was previously shown for similar reviews on public perceptions.^199^

However, we still concluded a few suggestions for how we can facilitate the comparison of different regions with each other and increase the positive perception of plant gene technologies. This brief list is by no means comprehensive. Rather, its purpose is to highlight and offer potential directions forward for the present and the future of plant gene technologies.

First, the research community should aim for a standardized assessment of public perceptions of plant gene technologies for different target groups to conduct comparative studies as has been done for other sensitive topics like evolution.^12^ This way, it would be possible to address how the various cultural backgrounds, as well as different legal situations, may lead to differences in public perception. The use of new genomic techniques in agriculture is controversial and its public acceptance, directly linked with consumers’ emotions, knowledge, lifestyle and beliefs,^19,22,117^ varies across different regions of the world. We did not, however, focus on the underlying sources of these factors, we only acknowledged that they exist. To enable a comparative and in-depth investigation of the factors predicting public acceptance, standardized measurement methods are required. The current state of research regarding public perception of plant gene technologies differs greatly between countries and regions in terms of the number of publications and survey instruments used. Many different methods have been used and different target groups have been addressed. Only a few studies directly compare public perception in different countries by the use of the same methods.^138,193^

Second, the engagement of scientists and experts in public debates about the future of GM products is crucial and may motivate scientists to take more action in public debates regarding the benefits of GM and GE products. During the research, scientists representing social sciences and humanities (SSH) should be involved. As highlighted in the EU Horizon 2020 program, communication of SSH research results is essential for ensuring impact on policy-making as well as for informing the broader public. Simis et al. (2016) proposed several solutions for how scientists and science communicators can continue moving science communication past the deficit model approach: i) training of science communication specialists who can deliver scientific information in a way that is understandable and engaging; ii) continuing communication training as part of education; science communication and public engagement should be a part of format training for researchers during the studies; iii) using a community-based approach to scientific problems and working with communities to answer their scientific questions.^200^ Conjointly, efforts should be made to use social media and popular blogs for reliable science communication about biotechnology in general and GM and GE food in particular.

Third, to improve awareness and understanding of plant gene technologies, it is important that scientists, policymakers and entrepreneurs create opportunities for the public to participate in relevant discussions and activities (e.g., citizen science projects). Moreover, these interactions facilitate monitoring changes in the acceptance of GE, GMOs, GM food and feed by the public. The development of biotechnology depends to a large degree on policymakers. Certainly, policy decisions are needed to clarify which genome-edited plants are covered by the current GM legislation. Unequivocally, both political influences and social acceptance, apart from scientific data, will significantly contribute to the future of GM/GE crops and food. During the past decade, countries of different world regions have already begun establishing regulatory criteria for new breeding techniques. While ethical standards and food security challenges are regionally specific, the legislation for genome-edited crops should follow scientific scrutiny. The advances in science and technology are enabling humanity to manage the challenges related to ensuring food security and decreasing natural resources on top of the COVID-19 pandemic. Changes in the acceptance of GE and GM food/crops must be monitored, and future research is required to meet the needs of society and to allow comparative analyses as well as causal links between legal requirements and public perceptions.

Finally, one of the ways to increase positive perception of GM food by consumers could be product labeling. Despite the opposition initially received from producer and farmer organizations, there is a strong indication that the new labels will not have adverse effects on the acceptance of GM food; in fact, the acceptance of GM food is predicted to grow once mandatory labels are implemented. However, caution is advised, as GM food labeling can cause counterproductive effects, especially in countries/regions where GM food acceptance is low. Therefore, to properly inform consumers, a well-thought-out GM food labeling campaign that provides reliable information about products should be planned and implemented.

## Supplementary Material

Supplemental Material

## Glossary

CRISPR/Cas9 (clustered regularly interspaced short palindromic repeats/Cas9): Cas9 (CRISPR-associated endonuclease 9) is an enzyme that binds guide RNA (gRNA) and uses it to recognize and cleave specific DNA sequences called CRISPR. It is one of the recently developed genome editing techniques.

Gene/genome editing (GE): The International Organization for Standardization (ISO) defines “genome editing” as “*techniques for genome engineering that involve DNA repair mechanisms and/or replication incorporating site-specific modification into a genomic DNA*.” ISO states that gene editing is a subclass of GE, without further indicating whether other subclasses are identified. Similarly, the use of the term “genome engineering” is considered here to relate to “genetic engineering” (see below) in the same way (ISO/DIS 5058-1). In this paper, when referring to the legal situation in countries where gene editing is not considered GMO, it concerns only those applications of gene editing where no foreign DNA has been added to the genome.

Genetic engineering: Term commonly used in the United States. The United States Department of Agriculture (USDA) defines genetic engineering as the “*manipulation of an organism’s genes by introducing, eliminating or rearranging specific genes using the methods of modern molecular biology, particularly those techniques referred to as recombinant DNA techniques.”*

Genetically modified organism (GMO): Term used in the European Union legislation, and defined in Directive 2001/18/EC, as “*an organism, with the exception of human beings, in which the genetic material has been altered in a way that does not occur naturally by mating and/or natural recombination*.”

SPR/Cas9 (c roup of recent technical innovations (molecular biology methods) allowing for the development of new plant varieties with desired traits. In the UE these new techniques are under discussion whether they result in a GMO under EU law and hence whether the resulting products fall under the scope of the current EU GMO legislation.

SPR/Cas9 (c ques capable of changing the genetic material of an organism and that have emerged or have been developed since 2001 when the existing GMO legislation in the EU was adopted.
