# Supplementary material for: Public perception of plant gene technologies worldwide in the light of food security
Source: GM Crops Food. 2022 Aug 22;13(1):218–41. doi: 10.1080/21645698.2022.2111946 (PMC9415543; doi:10.1080/21645698.2022.2111946)
Supplement: Supplemental Material [file KGMC_A_2111946_SM5659.docx]

Table S1. Comparative analysis of public perception of plant gene technologies across the world 2010 - 2021

(all references are below the Table)

| **Aspect studied** | **Europe** | **North America** | **Latin America** | **Asia** | **Africa** | **Australia and Oceania** |
| --- | --- | --- | --- | --- | --- | --- |
| attitude towards GM food/feed | 70% of Europeans agreed that GM food is fundamentally unnatural. 59% disagreed that GM food is safe for their health and that of their family [1]. 57% of Serbian said that GM foods negatively affect the health of people [2]. Enhanced shelf-life and quality were most often pointed out by Poles and British as benefits of GM foods (38% and 27% respectively) [3]. For Latvian inhabitants, GM feed was not safe for animal health as well as for human health [4]. 82% of Poles were afraid of GM foods [5]. In Denmark, 44% were against GM food [6]. The majority of Spanish was very positive about GM foods [7]*. In Italy, over half of the general public (N=1006) (54%) and the majority of scientists (81%) believed it is safe to eat GM foods [8]. | 49% of the US respondents have positive attitudes. 37% with neutral attitudes and 14% with negative attitudes towards GM science [9]. | In 2007, 50-60% of Costa Ricans did not think that GM crops pose risks to the environment. They thought that GM crops were nutritious. The absence of GM additives in oils was desirable for the majority of consumers (87%). A high proportion (73%) would accept oils with GM that reduces the use of pesticides [10]*.  66% of Brazilian students said that GM food was useful. For 78% of respondents, it was risky. 81% of Brazilian students said that GM crops are useful and for 63% are risky [11]*. 39% of Mexican teachers considered GM food to be hazards for future generations, however, 60% believed that they would be useful in preventing world hunger [12]. | Only 6% of Korean consumers had a positive stance on GM foods, while 59% perceived GM foods as being risky to human health [13]. 24% of the Turkish students thought that GM foods were extremely risky. 37% of Turkish consumers thought that GM foods did not pose a hazard t4]*. In Pakistan, 61% of students heard about GM food; 48% have correctly defined GM foods; students were not aware of the availability of GM foods in Pakistan (69%) and had an opinion that genetic modification has made the quality of life better (55%) [15]. About 40% of China consumers perceived GM foods as safe [16]. | In Kenya more than 80% agreed that GM technology increases productivity [17]*. 67% of respondents in Kenya agreed that using GM technology in food production increases productivity and offers a solution to the world food problem [18].Thirty percent of Nigerian students had no idea about the applications of biotechnology [19]. | 75% of respondents in Australia had heard about GM food, and 25% had not. [20]*. 68% of respondents from Australia thought GM foods are an important food issue [21]. |
| acceptance of the use of biotechnology in food production | 61% of Europeans disagreed that the development of GM food should be encouraged. Europeans had strong reservations about animal cloning in food production and did not see the benefits, and felt that it should not be encouraged [1]. | 58% of US respondents agreed that the application of biotechnology in food should be encouraged. 69% said that biotechnology in crops should be encouraged [22]*. | Approx. 31% of Costa Ricans were supportive of research related to GM crops. 25% disagreed, and the remaining 44% did  not know or either had not heard about GM crops [10]*.  About 62% of respondents in Jamaica were against the use of genetic engineering to create or enhance the taste of fruits and vegetables [23]*. | Less than 40% of respondents from Turkey said that biotechnology applications for producing GM foods were useful for society [24]*. | Most of the Kenyan respondents agreed (73%) or strongly agreed (9%) that GM crops can increase productivity [25]*.  46% of respondents from cities and rural areas agreed that GM food should be legalized in Tanzania and farmers allowed to grow them immediately, whereas 24% disagreed [26]. | More than half of Australian high school students supported GM plants but less than one-third accepted animal biotechnology [27]*. |
| acceptance of the use of biotechnology in medicine | 50% of Poles supported the medical use of GMOs in the production of drugs/vaccines or life-saving procedures [5]. 55% of Dane had a positive attitude to genetic modification for medical purposes [6]. | 49% of US respondents agreed that the application of biotechnology in cloning should be encouraged. 78% said that biotechnology in medicine should be encouraged, and 83% said that biotechnology in tests for hereditary diseases should be encouraged [22]*. | 87% of Brazilian students said that genetic modification for medical applications was useful [11]*. More than three-quarters of respondents in Jamaica favored the use of genetic engineering to create or enhance new drugs [23]*. | The majority of respondents in Turkey supported the use of biotechnology for disease treatment (94%) and medicine production (86%) [24]*. 69% of Japanese participants favoured the promotion of genomic studies related to medicine [28]. | no data | Australian high school students were highly supportive of biotechnology for engineering micro-organisms and humans [27]*. |
| willingness to eat/buy GM food | 74% of Bosnians were afraid of consuming GM food [29]. The majority of Serbian (52%) demonstrated negative attitudes towards consuming and buying GM food [2]. The majority of Italians were not willing to buy GM food products even if they have nutritionally enhanced benefits [30-31]. About 49% of French consumers (N=1109) were willing to purchase biotechnology-produced fresh fruit [32]. | 56% of US respondents would consume both GM and CRISPR food. 21% would not consume either, 15% would consume CRISPR only, and 8% GM only [33]. 74% of respondents in the US and Canada would consume food produced with RNAi and Bt technologies [34].  More than 56% of respondents of US consumers were willing to buy and consume both GM and CRISPR food. Among those who had preferences, respondents were more willing to consume CRISPR than GM food [33]. | In Costa Rica, 40-50% of respondents would buy GM food, in Jamaica ~50% [10*,23]. 35% of Mexican teachers thought that eating GM food will be harmful to their health and their family’s health [12].  Costa Ricans would consume GE foods if the nutritional  quality were higher (71%) and if they were cheaper than  conventional products (61%). 59% responded positively to consuming GE agricultural products if they were available in the national market. 55% of Costa Ricans said that they would consume it if it had the same price as conventional products [35]. | Only 25% of Chinese urban consumers and 29% of scientists were willing to buy GM soybean oil [36]. 41% of Indian students were willing to buy GM food [37]. 55% of Korean consumers said they would avoid purchasing GM foods in the future [17]. About 36% of South Koreans (N=1003) were willing to purchase biotechnology-produced fresh fruit [32]. 79% of China consumers indicated they intend to purchase GM foods [16]. | In Uganda, 73-77% of respondents and 68% in Kenya would buy GM maize. 92% of respondents in Uganda would buy GM banana if it was more nutritious. 43% of South Africans agreed that GM crops are not safe to eat [38-39, 17, 25, 40]. | 10% of respondents from New Zealand had an intention to purchase food produced using genetic engineering. 30% had no purchasing intentions and 60% had a (strong) intention not to purchase [41]*. 63% of respondents in New Zealand were willing to pay more for the GM apples that were better for the environment. 56% wanted to pay more for the GM apples that had a health benefit [42]*. 70% of Australians were willing to eat BT-rice treated with RNAi [34]. 51% would consume both GM and CRISPR-rice [33]. 61% of farmers from West Australia were willing to consume flour from GM wheat that is higher yielding. 57% were willing to consume it when resistant to pests/herbicides [43]*. |
| perception of benefits of genome editing | In Norway, respondents saw the benefits of using GE on crop plants, such as reducing pesticides and crop loss (68%), adapting a crop plant to a changing climate (65%), and improving the nutritional content of a crop plant (52% ) [44]. | 36% of Americans expected that the benefits for society would outnumber the downsides of gene editing [45]. | The majority of Costa Ricans agreed that GE would increase crop production in the country (66%);  improve the economy (64%);  bring benefits to respondents’ families (61%);  bring benefits to the environment (57%) [35]. | no data | no data | no data |
| risks/concerns related to genome editing | 60% of Norwegians were worried that gene-edited products present risk to health or the environment [44]. 46% of respondents in the UK were wary of potential risks of genome editing [46]. 61% of respondents in Spain agreed that science and technology is a source of risk [47]. 4% of Europeans are concerned about GE [48]. | no data | In 2019 over one-third of respondents in Costa Rica perceived a medium or high risk of GE to the quality of life, health and environment [35]. | Malaysians perceived modern biotechnology as moderately risky to human health [49-50]. | no data | no data |
| risks/concerns related to GM food, biotechnology | 59% of Europeans said that the application of modern biotechnology in food production is risky [51]*. | About 24% of US adults said GM foods were very likely to lead to problems for public health, while 21% said it’s very likely that these foods will create problems for the environment [45].  About half of Americans saw no difference between GM and other foods, while a sizable minority said GM foods are a health risk [45]. More Americans considered the risk of eating GM foods either medium (15% of all US adults) or high (20% of all US adults) than consider the health threat of GM foods to be low (just 4% of all US adults) [45]. | 78% and 63% of Brazilian high school students believed that genetic modification of food and crops, respectively, is risky [11]*. 21% of Costa Ricans expressed fear that biotech-derived food had any health risks [10]*. Over half of respondents from Paraguay believed that genetically engineered crops are dangerous [52]. | In Korea, 59% of consumers perceived GM food as risky to human health [13]. In Turkey, 24% thought of GM food as extremely risky, 69% as little risky [14]*. 35% of Chinese internet users perceived GM food as risky [53]. Most Chinese agribusiness managers were concerned about GM foods and opposed their application [54]. Nearly 56% of China consumers were concerned about the health effects of GM foods; 58% of consumers were concerned about the environmental effects of GM foods [16]. | 35% of Tanzanians agreed that the risks from GM food were acceptable, while 26% disagreed [26]. In Kenya, 50% of consumers feared that GM crops technology can lead to a loss of traditional maize varieties and affect untargeted insects (51%); 37% feared that consumption of GM foods can damage one’s health or cause allergic reactions (40%) [17]*. | One in three Australian agricultural professionals believed that the benefits of genetic engineering did not outweigh the costs/risks [55]. 62% of respondents from Australia were concerned about GMOs in food [56]. |
| trust in particular players | For Latvian respondents, the information provided by the media on GM crops is untrustworthy and unreliable (64%) [4]. 47% of Serbians had no trust in state authorities regarding GM foods, 42% in science and 31% in NGOs, the highest distrust (74%) was directed toward foreign producers of GM food [2]. Dutchmen had a relatively high level of trust in GM regulations [57]. | 44% of respondents from the US said that the media did a good job for society; 58% said the same for industry, 47% for ethics committees, 59% for the consumer organisations, 51% for environmental groups, 40% for government, 59% for shops, 73% for farms, 73% churches and 59% for doctors [22]*.  Americans were somewhat skeptical of information from scientists. A minority of 35% said they trust  scientists a lot to give full and accurate information about  the health effects of eating GM foods. About one-in-five said  they do not trust information from scientists at all or not too much. Another 43% of US adults reported some trust in scientists’ information.  A similar share of Americans trusted small farm owners a lot (29%) or some (49%) to give full and accurate information  about the health effects of GM foods. No more than one-in-ten Americans trusted each of these groups a lot - food industry leaders and elected officials [45].  In the US trust was highest in university scientists and farmers and lowest in grocers and grocery stores and food  manufacturers [58]. | no data | Nearly 60% of Chinese consumers did not trust GM scientists. In total 42% of Chinese consumers trusted the government and 39% trusted non-GM scientists or individuals. Around 35% of consumers believed misinformation on GM technology that was provided by the media. Around 50% of respondents from China had no confidence in the government's management of biotechnology, while only 17% had confidence [54, 59]. Most China consumers (72%) trusted the agency overseeing GMO safety [20]. | The trust of Ugandan consumers for control of GM crop release was highest in local leaders (78%) and some ministries (NEMA 89%), followed by scientists (NARO 73%, university 66%). Trust in NGOs and food processors was lower (62% and 41% respectively) [40]. | Researchers received trust from >80% of students while government, farmers and environmental organisations scored low (25-30%).  Less than 20% of high school students from the rural Riverina region of New South Wales, Australia, trusted the press and internet [60]*. |
| labeling | The rate of support for mandatory labeling of GM products varied from 76% to 92% in various European countries (76% in Norway, 81%-92% in Slovenia, 92% in Poland, 87-91% in Spain, 85% in Serbia) [44, 61*, 5, 7, 62]. 64% said that the government in Bosnia and Herzegovina should allow GM food [29]. | 92% of US consumers believed that before genetically engineered food can be sold, it must be  labeled accordingly [63]. 89% of the US said they favor mandatory labels on “foods which have been genetically engineered or containing genetically engineered ingredients be labeled to indicate that.” [64]. | 96% of Jamaican respondents agreed or strongly agreed that foods produced through genetic engineering should be labeled [23]*. Brazilian high school students were strongly supportive of the labeling of transgenic foods [11]*. | 80% of Turkey residents wanted to have labeling on GM food [14]*. Koreans were willing to pay extra for GMO labeling policies [65]. In Pakistan, 70% of students said that the food should be accurately labeled [15]. About 89% of China consumers considered the “contains GMO” label as very and moderately important [16]. | In South Africa, 75% of respondents agreed with the statement that food products containing GM should be labeled [21]. 46% of respondents said that GM food should be legalized in Tanzania and farmers are allowed to grow them immediately [66]. | no data |

* In some studies, due to a lack of current data the authors showed results from studies conducted before 2010.

GM - genetically modified

GMO - genetically modified organism

**References:**

[1] Eurobarometer 73.1. (2010). Available from https://data.europa.eu/data/datasets/s755_73_1_ebs341?locale=pl [accessed 16 May 2021]

[2] Brankov, T.P. et al. (2013) The impact of biotechnology knowledge on the acceptance of genetically modified food in Serbia. Rom. Biotech. Lett. 18(3), 8295 - 8306

[3] Popek, S. and Halagarda, M. (2017) Genetically modified foods: Consumer awareness, opinions and attitudes in selected EU countries. Int. J. Consum. Stud.41, 325 - 332. https://doi.org/10.1111/ijcs.12345

[4] Aleksejeva, I. (2016) An Empirical Study of Latvian Consumers' Attitudes and Perceptions Towards Genetically Modified Organisms. European Integration Studies 10, 157-168. DOI:10.5755/j01.eis.0.10.14624

[5] Rzymski, P. and Królczyk, A. (2016) Attitudes toward genetically modified organisms in Poland: to GMO or not to GMO? Food Secur. 8, 689 - 697. DOI:10.1007/s12571-016-0572-z

[6] Mielby, H. et al. (2013) The role of scientific knowledge in shaping public attitudes to GM technologies. Public Underst. Sci. 22(2), 155-168. https://doi.org/10.1177/0963662511430577

[7]* Ramon, D. et al. (2008) Food biotechnology and education. Electron. J. Biotechnol. 11(5), 1-5.

[8] Pappalardo, G., D’Amico, M., and Lusk, J.L. (2021) Comparing the views of the Italian general public and scientists on GMOs. International Journal of Food Science & Technology 56(7), 3641-3650. DOI: 10.1111/ijfs.14993

[10]* Espinoza-Esquivel, A. M., and Arrieta-Espinoza, G. (2007) A multidisciplinary approach directed towards the commercial release of transgenic herbicide-tolerant rice in Costa Rica. Transgenic Res. 16(5), 541-555. DOI:10.1007/s11248-007-9068-0

[11]* Massarani, L., and Moreira, I. D. C. (2005) Attitudes towards genetics: a case study among Brazilian high school students. Public Underst. Sci. 14(2), 201-212. DOI:10.1177/0963662505050992

[12] Jiménez‐Salas, Z. et al. (2017) Basic‐education mexican teachers' knowledge of biotechnology and attitudes about the consumption of genetically modified foods. Biochem. Mol. Biol. Educ. 45(5), 396-402. DOI:10.1002/bmb.21058

[13] Kim, N.H. et al. (2018) Strategic approaches to communicating with food consumers about genetically modified food. Food Control 2018; 92:523-53 https://doi.org/10.1016/j.foodcont.2018.05.016.

[14]* Basaran, P. et al. (2004) Public perceptions of GMOs in food in Turkey: A pilot survey. J. Food Agric. Environ. 2, 25-28

[15] Amin, R. et al. (2021) Knowledge and attitudes toward genetically modified (GM) food among health sciences university students in Karachi, Pakistan. Nutrition &Food Science, 51(7), 1150-1162. https://doi.org/10.1108/NFS-01-2021-0019

[16] Zheng, Q. and Wang, H.H. (2021) Do Consumers View the Genetically Modified Food Labeling Systems Differently? “Contains GMO” Versus “Non-GMO” Labels. The Chinese Economy 54(6), 376-388. https://doi.org/10.1080/10971475.2021.1890356

[17]* Mugo, S. et al. (2005) Developing Bt maize for resource-poor farmers - Recent advances in the IRMA project. Afr. J. Biotechnol. 4(13),1490-1504

[18] Bett, C. et al. (2010) Perspectives of gatekeepers in the Kenyan food industry towards genetically modified food. Food Policy 35, 332-340

[19] Kooffreh, M.E., Ikpeme, E.V., and Mgbado, T.I. (2021) Knowledge, perception, and interest regarding biotechnology among secondary school students in Calabar, Cross River State, Nigeria. Biochem Mol Biol Educ. 49(4), 664-668. DOI: 10.1002/bmb.21507

[20]* James, S., Burton, M. (2003) Consumer preferences for GM food and other attributes of the food system. The Australian Journal of Agricultural and Resource Economics, 47(4), 501-518

[21] Meyer, S.B. et al. (2014) The importance of food issues in society: Results from a national survey in Australia. Nutrition & Dietetics, 71(2),108-116

[22]* Priest, S. H. et al. (2003) The ‘trust gap’ hypothesis: Predicting support for biotechnology across national cultures as a function of trust in actors. Risk Anal. 23(4), 751-766. DOI:10.1111/1539-6924.00353

[23]* Abdulkadri, A. O. et al. (2004) Public perception of genetic engineering and the choice to purchase genetically modified food. Materials from the American Agricultural Economics Annual Meeting, Denver, Colorado, August 1-4. DOI:10.22004/ag.econ.19984

[24]* Demirci, A. (2008) Perceptions and attitudes of geography teachers to biotechnology: A study focusing on genetically modified (GM) foods. Afr. J. Biotechnol. 7(23), 4321-4327

[25]* Kimenju, S.C. and Degroote, H. (2008) Consumer willingness to pay for genetically modified food in Kenya. Agric. Econ. 38, 35–46. https://doi.org/10.1111/j.1574-0862.2007.00279.x

[26] Mnaranara, T.E. et al. (2017) Public perception towards genetically modified foods in Tanzania. J. Anim. Pl. Sci. 27, 589–602

[27]* Dawson, V. (2007) An exploration of high school (12-17 year old) students' understandings of, and attitudes towards biotechnology processes. Res. Sci. Educ. 37(1), 59-73. DOI:10.1007/s11165-006-9016-7

[28] Ishiyama, I. et al. (2009). Relationship between public attitudes toward genomic studies related to medicine and their level of genomic literacy in Japan. Am. J. Med. Genet. A. 146(13), 1696-1706. DOI:10.1002/ajmg.a.32322

[29] Bevanda, L. et al. (2017) Public opinion toward GMOs and biotechnology in Bosnia and Herzegovina. In CMBEBIH 2017. IFMBE Proceedings (Badnjevic A., eds), 62, Springer, Singapore. https://doi.org/10.1007/978-981-10-4166-2_70

[30] Montuori, P. et al. (2012) The consumption of genetically modified foods in Italian high school students. Food. Qual. Prefer. 26(2), 246-251. DOI:10.1016/j.foodqual.2012.05.004

[31] Canavari, M., and Nayga, R.M. (2009) On consumers' willingness to purchase nutritionally enhanced genetically modified food. Appl. Econ. 41(1), 125-137. DOI:10.1080/00036840701367564

[32] Heng, Y., Yoon, S., and House, L. (2021) Explore Consumers’ Willingness to Purchase Biotechnology Produced Fruit: An International Study. Sustainability 13(22), 12882. https://doi.org/10.3390/su132212882

[33] Ruth T.K. et al. (2019) Are American's Attitudes Toward GM Science Really Negative? An Academic Examination of Attitudes and Willingness to Expose Attitudes. Sci. Commun. 41(1), 113-131. DOI:10.1177/1075547018819935

[34] Shew, A.M. et al. (2017) New innovations in agricultural biotech: Consumer acceptance of topical RNAi in rice production. Food Control 81, 189e195. https://doi.org/10.1016/j.foodcont.2017.05.047

[35] Gatica-Arias, A. et al. (2019) Consumer attitudes toward food crops developed by CRISPR/Cas9 in Costa Rica. Plant Cell Tiss Org. 139(2), 417-427. DOI: https://doi.org/10.1007/s11240-019-01647-x

[36] Huang, J.K. et al. (2017) Scientists' attitudes toward agricultural GM technology development and GM food in China. China Agric. Econ. Rev. 9(3), 369-384. DOI:10.1108/CAER-05-2017-0101

[37] Kajale, D.B. and Becker, T.C. (2013) Determinants of consumer support for mandatory labeling of genetically modified food in India - A student survey. Br. Food J. 115(11), 1597-1611. DOI:10.1108/BFJ-12-20110302

[38] Wamatsembe, I.M. et al. (2015) A Survey: Potential Impact of Genetically Modified Maize Tolerant to Drought or Resistant to Stem Borers in Uganda. Agronomy 7, 24.

[39] Guenther, L. and Joubert, M. (2018) Support for research in climate change and nuclear energy, but less so for fracking: Bornfree South Africans’ attitudes towards scientific controversies. Afr. J. Sci. Technol. Innov. Dev. 10(1), 2042-1338. DOI:10.1080/20421338.2017.1399535

[40] Kikulwe, E.M. et al. (2011) Attitudes, perceptions, and trust. Insights from a consumer survey regarding genetically modified banana in Uganda. Appetite 57, 401–413

[41]* Cook, A.J. et al. (2002) Attitudes and intentions towards purchasing GM food. J. Econ. Psychol. 23(5), 557-572. DOI:10.1016/S0167-4870(02)00117-4

[42]* Kassardjian, E. et al. (2005) A new approach to elicit consumers’ willingness to purchase genetically modified apples. Br. Food J. 107(8), 541-555. DOI:10.1108/00070700510610968

[43]* McDougall, D.J. et al. (2001) Attitudes of pulse farmers in Western Australia towards genetically modified organisms in agriculture. Australas. Biotechnol. 11(3), 36-39

[44] Norway Report (2020). Available from https://www.bioteknologiradet.no/filarkiv/2020/04/Report-consumer-attitudes-to-gene-editing-agri-and-aqua-FINAL.pdf [accessed 15 June 2021]

[45] Pew Research Center (2016) U.S. public opinion on the future use of gene editing. Available form https://www.pewresearch.org/science/2016/07/26/u-s-public-opinion-on-the-future-use-of-gene-editing/ [accessed 15 June 2021]

[46] Van Mil A. et al. (2017) Potential uses for genetic technologies: dialogue and engagement research conducted on behalf of the Royal Society: Findings Report. Available from https://royalsociety.org/~/media/policy/projects/gene-tech/genetic-technologies-public-dialogue-hvm-full-report.pdf [accessed 25 May 2021]

[47] Costa-Font M., Gil J.M. (2012). Meta-attitudes and the local formation of consumer judgments towards genetically modified food

[48] Food Safety Report (2019). Available from https://www.efsa.europa.eu/sites/default/files/corporate_publications/files/Eurobarometer2019_Food-safety-in-the-EU_Full-report.pdf [accessed 15 June 2021]

[49] Amin, L. et al. (2011) Ethical perception of cross-species gene transfer in plant. Afr. J. Biotechnol. 10(58), 12457-12468

[50] Amin, L. et al. (2009) Ethical Dimensions of Modern Biotechnology in Malaysia. EDU '09: Proceedings of the 8th WSEAS International Conference on Education and Educational Technology, Edited by: Revetria, R., Mladenov, V., Mastorakis, N. Book Series: Recent Advances in Computer Engineering

[51]* Eurobarometer 52.1 (2000). The Europeans and biotechnology in 1999. Available from https://www.gesis.org/en/eurobarometer-data-service/survey-series/standard-special-eb/study-overview/eurobarometer-521-za-3205-nov-dec-1999 [accessed 25 May 2021]

[52] Candia, N.B. et al. (2021) Perception of genetically engineered crops in Paraguay. GM Crops & Food 12(1), 409-418. https://doi.org/10.1080/21645698.2021.1969835

[53] Zhang, Y.Y. et al. (2018) Application of an integrated framework to examine Chinese consumers' purchase intention toward genetically modified food. Food. Qual. Prefer. 65, 118-128. DOI:10.1016/j.foodqual.2017.11.001

[54] Deng, H.Y. et al. (2017) Attitudes toward GM foods, biotechnology R&D investment and lobbying activities among agribusiness firms in the food, feed, chemical and seed industries in China. China Agric. Econ. Rev. 9(3), 385-396. DOI:10.1108/CAER-10-2016-0162

[55] Wheeler, S.A. (2009) Exploring the influences on Australian agricultural professionals' genetic engineering beliefs: an empirical analysis. J. Technol. Transf. 34(4), 422-439. DOI:10.1007/s10961-008-9094-y

[56] Mohr, P. and Golley, S. (2016) Responses to GM food content in context with food integrity issues: results from Australian population surveys. New Biotechnol. 33(1). 91-98. DOI:10.1016/j.nbt.2015.08.005

[57] Hanssen, L. et al. (2018) Revisiting public debate on Genetic Modification and Genetically Modified Organisms. Explanations for contemporary Dutch public attitudes. JCOM-J.Sci. Commun. 17(04). A01. https://doi.org/10.22323/2.17040201

[58] Lang, J. T. (2013) Elements of public trust in the American food system: Experts, organizations, and genetically modified food. Food Policy 41, 145-154. DOI: 10.1016/j.foodpol.2013.05.008

[59] Yu, C.X. et al. (2020) Attitude Gaps with Respect to GM Non-Food Crops and GM Food Crops and Confidence in the Government's Management of Biotechnology: Evidence from Beijing Consumers, Chinese Farmers, Journalists, and Government Officials. Sustainability 12(1), 324. DOI:10.3390/su12010324

[60]* Cavanagh, H. et al. (2005) Riverina high school students’ views of biotechnology. Electron. J. Biotechnol. 8 (2). DOI:10.4067/S0717-34582005000200001

[61] Plahuta, P. et al. (2007) Slovenian public opinion regarding genetically modified organisms in winemaking. Acta Aliment. 36(1), 61-73. DOI:10.1556/AAlim.36.2007.1.8

[62] Nonic, M. et al. (2014) State and Perspectives of Genetically Modified Trees in Some Western Balkan Countries. Tree Biotechnology. Edited by Ramawat K.G., Merillon J.M., Ahuja M.R. 366-391

[63] Consumer Reports (2014) Available form http://consumersunion.org/news/new-consumer-reports-poll-shows-consumer-demand-for-strong-federal-standards-for-genetically-engineered-food/ [accessed 26 June 2021]

[64] The Mellman Group, Inc. (2015) Available from https://www.centerforfoodsafety.org/issues/976/ge-food-labeling/us-polls-on-ge-food-labeling#:~:text=http%3A//4bgr3aepis44c9bxt1ulxsyq.wpengine.netdna-cdn.com/wp-content/uploads/2015/12/15memn20-JLI-d6.pdf [accessed 26 June 2021]

[65] Lee, J.S., and Yoo, S.H. (2011) Willingness to pay for GMO labeling policies: the case of Korea. J. Food Saf. 31, 160-168. https://doi.org/10.1111/j.1745-4565.2010.00280.x

[66] Gastrow, M. et al. (2016) Public Perceptions of Biotechnology in South Africa. http://biosafety.org.za/information/know-the-basics/gmos-and-society/public-perceptions-of-biotechnology-in-south-africa-
